# Visceroptosis

**Published:** 1912-08

**Authors:** J. Norment Baker

**Affiliations:** Montgomery, Ala.; Secretary of the Medical Association of the State of Alabama; Surgeon to St. Margaret’s Hospital


### VISCEROPTOSIS.

By J. Norment Baker, B. A., M. D., Montgomery, Ala.

*Secretary of the Medical Association of the State of Alabama; Surgeon to St. Margaret's Hospital.*

More than twenty-five years ago, Glenard, the father of visceroptosis, spoke these words:

"I can affirm," he says, "that the physician who will follow my directions and strive to verify my statements in such cases (meanings visceroptics) will find in his practice the satisfaction which a *positive* diagnosis gives to both physician and patient, from which alone a proper prognosis can be made; and that satisfaction, the greatest of all, which directs the treatment and avoids for the patient the trial upon him of so many remedies, while at the same time it secures him relief and prevents the physician himself from falling into therapeutic skepticism."

These words are little short of prophetic, and are just as true, or more true, to-day, than they were when uttered by Glenard, for the reason that the gauntlet now run by the poor patient is more thickly studded with pseudo-surgeons who stand ready to "anchor" everything "loose," even to the patient's morals.

The term first used by Glenard was *enteroptosis*, or a displacement of the intestine; his conception being that the "fons et origo" of the whole disorder was a sagging of the hepatic flexure of the colon. Starting with this theory as his foundation stone, he proceeded to weave, with enviable enthusiasm, a most elaborate superstructure, bringing into it that medley of symptoms and that peculiar physical form which to-day we recognize as characterizing the enteroptotic.

The term, *visceroptosis,* is a more inclusive one and embraces within its compass a looseness or sagging of any viscus, either abdominal or pelvic; if, however, one cares to be more specific, this can be done by adding, to the organ affected, the word *ptosis,* as gastroptosis, nephroptosis, coloptosis, and the like.

*Etiology.*—The study of visceral ptosis, from an etiologic standpoint, has provoked a variety of theories differing so widely that it is manifestly impossible to find one theory broad enough to explain all cases.

Stiller holds that it is a congenital anomaly; unquestionably, the factor of heredity plays a most potent part and is not to be lightly dealt with. Noble, of Philadelphia, in his sweeping hypothesis of "hereditary hypoplasia" gives to the careful student much food for thought. According to his theory, this hereditary hypoplasia prominently manifests itself at or about the age of puberty—a time when the normal girl discards the characteristics of the neuter or child and assumes the form and figure of the essentially feminine. In those cases stigmatized by this inherent tendency to a lack of development, we find the age of puberty much delayed; the thyroid and mammary glands fail to develop; the hips fail to broaden. Not alone are these gross external features absent, but the uterus, with its ligaments and supports, the ovaries, the tubes and their ligaments, all, still bear the earmarks of the child; the cervix is long and represents the "pinhole os" of Marion Sims. In short, we see in this type of creature an absolute inability to respond, by a normal physiologic hypertrophy to the first serious demand made upon it by nature. This developmental failure is not limited to the reproductive organs alone; it permeates every organ and every tissue of the whole being, because it is inherent and is an integral part of the fertilized ovum from which the creature had its beginning. That is to say, there is a general hypoplasia of the entire body—muscular, nervous, visceral.

Stiller has found that in from 80 to 90 per cent. of all cases, a typical constitution of the patient could be found; being the anatomic substratum for the clinical symptom-complex designated by him as "congenital universal asthenia;" that is to say a congenital constitutional weakness. We are able now to recognize this constitutional asthenia by the peculiar structure of the thorax and abdomen.

The so-called "habitus enteropticus" is a true anatomic basis for the whole symptom-complex. Given this type of woman, thus seriously handicapped by nature, and heap upon her the burdens of a strenuous and laborious life, the wear and tear of frequent child-bearing, with its conseqeunt relaxation of the abdominal musculature, and it is not difficult to picture the result as being a creature with a ptosis of every viscus within her abdomen and pelvis.

Further, a careful analytical study of the history, both personal and family (meaning by this that we delve deeply and painstakingly into the forbears of the patient of a victim of enteroptosis, will often divulge facts of the first importance; it will be ascertained that such a sufferer is a member of a family in a period of involution and that she is the luckless survivor, or at least one of the few luckless survivors of a family about to become extinct, and that here are present in her physical form unmistakable evidences of degeneracy and physical decline.

Keith takes the view—which when carefully analyzed, has much to commend it—that enteroptosis results largely from a vitiated method of respiration; meaning that faulty movements of the diaphragm, resulting from various causes, as will be explained a little later, determine visceral ptosis.

Quite recently, Longyear, of Detroit, has presented a very attractive theory which seeks to explain these ptoses by a pull which is exerted primarily on the kidney, through the nephrocolic ligament, by the sagging of the large bowel. With this descent of the large bowel and kidney comes also an involvement of the duodenum, and subsequently the stomach. He also contends that the prolapse of the kidney is always secondary to the drop in the colon, for the reason that many cases show, with the radiograph, ptosis of the large bowel without kidney involvement, while in no case could a renal ptosis be demonstrated which had not also a coloptosis.

In order to gain a clearer conception of the general subject of visceroptosis, one must recall to mind the various factors which are instrumental in retaining the various abdominal organs in position. There exists within the abdominal cavity a definte hydrostatic pressure which exerts a greater or less force in the maintenance of these various organs in their proper location; should the dimension of this cavity be in any way altered, this intra-abdominal pressure likewise undergoes an alteration and the organs are likely to yield to this decrease in pressure and sagging will be the consequence.

The effect of posture is probably of even greater importance than that of hydrostatic pressure. The upright position—that is, the one in which the body is made as tall as possible without rising on the toes—is the attitude in which there is least strain and is, therefore, the correct attitude. In such position the muscles of the trunk are in such balance that while the anterior and posterior groups are in slight contraction, none are strained and but few of the ligaments are under tension. This holds true alike for all three of the important cavities of the trunk—theracic, abdominal and pelvic. The heart and lungs are less incommoded; the anterior abdominal muscles are put on a stretch, thereby securing a tone which exerts an even pressure on the abdominal and pelvic contents and thus tends to keep each viscus within its proper sphere.

If, therefore, the erect poise be the proper one from the viewpoint of economic functional efficiency, any marked deviation therefrom must, of necessity, produce disturbances in visceral support. If the body be habitually inclined forward, the chest is flattened above and the normal, free action of the heart and lungs may be materially embarrassed. Thus it is that we most often find that in a patient who has the "habitus enteropticus" well established, there is a pronounced drooping of the shoulders, with a chest undersized, collapsed and flattened. The intercostal spaces above are widely separated, while the lower ribs are crowded closely together and slant markedly downward and inward, producing a very deep epigastric angle which is quite characteristic.

The capacity of the upper or thoracic abdomen bears an indirect ratio to the depth of this epigastric angle; that is to say, the deeper and more acute this angle becomes, the less capacious will be this region. Indeed, the size and form of the thorax and upper abdomen as seen and estimated, serve as a fair index to the position of the abdominal viscera.

Further, it is but an easy corollary to trace how this sagging and giving away of the bony framework of the thorax will materially interfere with the proper functioning of that very important structure, the diaphragm. The drooping forward of the chest necessarily shortens the vertical diameter of the abdomen, with, as a consequence, an increase in the antero-posterior diameter and a stretching of the diaphragm, also anteroposteriorly, which produces a lowering of the dome or arch of this cavity.

This is the mechanical explanation of Keith's theory of enteroptosis as being a vitiated method of respiration.

Again, let us trace the inevitable consequences which must, per force, befall those abdominal viscera swung from and immediately adjacent to, such a diaphragm—notably the liver, stomach, transverse colon, duodenum and kidneys—more especially the right.

The concave lower surface of the diaphragm fits the upper convex aspect of the liver like the cover of a baseball, and just so much as the former is displaced, by just so much will the latter be displaced. Hence the frequent prominent anterior border of the liver in these subjects. It is easy to see also, how such a displacement might, by the production of "kinks" in various portions of the bile passages, lead to bile stasis, infection and consequent cholelithiasis; thus adding a real pathological lesion as ground for explanation of much of the dyspeptic distress so often encountered.

The stomach owes its stability of position largely to the attachment of the esophagus to the diaphragm, plus its own mesentery; consequently a downward droop of the diaphragm produces the same effect upon the stomach. With this downward movement the chief sag takes place first in the lower or pyloric half of the stomach, this being the most dependent portion. Since, however, the possible downward movement of the pyloric end is limited by the firm attachment of the second and third portions of the duodenum—these, it being recalled, are retroperitoneal and have but very limited motion—the amount of sag at the pylorus is not great, but the rest of the organ can sag downward to almost any degree. To permit this, the upper portion of the stomach, which is naturally the largest, becomes elongated and stretched, while the large or prominent part of the organ develops in the middle, or much lower down than is normal. As the stomach elongates, taking with it the first or movable portion of the duodenum, we thus see that more or less angulation or kinking occurs in the duodenum which materially embarrasses proper gastric drainage. These factors, varying with the degree of elasticity of the individual observer's imagination, can explain as many or as few of the digestive ailments as he may see fit.

The whole of the small intestine, too, is forced downward by the displacement from above and the circulation and innervation of this segment of the digestive tract is impaired, as well as the absorptive capacity being seriously crippled.

With the large bowel somewhat similar conditions exist, but it must be remembered that usually, the ascending colon has no mesentery from just above the cecum to the hepatic angle, where the transverse colon begins; this latter has a complete menenteric covering which suspends it from the lower border of the stomach; at the splenic flexure it again loses its mesentery, becoming retroperitoneal as the descending colon. Thus it is seen that the large bowel is more or less fixed at the two points of angulation, that is, at the hepatic and splenic flexures, and that the transverse portion, attached to the stomach, will sag and droop just in proportion as the stomach does.

Here again we have the free movement of the fecal current materially hampered and a ready explanation offered for the intractable constipation which usually marks these sufferers. It is by no means an unusual picture to find in these cases a transverse colon well down in the pelvis.

Numerous workers, experimenting with the X-ray in these cases by means of the bismuth paste meal, have been able to demonstrate not only the *transverse* colon, but practically the whole of the large bowel, occupying the pelvis, with, as may well be supposed, much kinking, crumpling and angulation of this bowel.

With this colonic and visceral descent (even though the nephrocolic ligament as described by Longyear, be not such an important factor as he would have us believe) together with a general disturbance of the intra-abdominal pressure, it requires no great stretch of the imaginaion to trace how the kidneys, too, might clamor for and gain recognition in, this general descensus. The right kidney suffers most often, for the reason that the tug and pull are exerted almost entirely on viscera with which it is most intimately associated.

*Symptomatology.*—Visceroptosis does not invariably give rise to symptoms, and we should clearly recognize the fact that enteroptosis and good health, as ordinarily considered, are not incompatible. We not infrequently see about us women of a very marked type who are apparently enjoying excellent health; they feel well, are free of pain, and are able to take their places in the social world.

Yet while this may be true, it should likewise be remembered that the physical equilibrium and mental poise of these patients is ever an uncertain equation; overwork, frequent child-bearing, sudden severe shocks, unhappy domestic environments—any or all of these—may serve to destroy the balance and to precipitate a frightfully complex medley of symptoms.

These symptoms are, as a rule, fairly well defined, though they may be as protean and as complicated as the manifestation of syphilis. Most often, those referable to the digestive and reproductive organs are most prominent, together with a train, of greater or less length, largely psychoneurotic in character; such as a vague and indefinite pain in the back, loins and groin; heavy distressful feelings in the upper abdomen, and dragging, pulling sensation in the pelvis; constipation and flatulence also are rarely absent and are usually marked. Throbbing in the abdomen and aortic pulsation are often to be found.

The physical form, as a whole, gives very decided impression of weakness and frailness and the facial expression oft-times bespeaks a "neurasthenia basis." She (for these patients are nearly all females) is slender and angular, with no compactness of form and with marked paucity of adipose tissue. The whole muscular system is poorly developed—flabby, flaccid and lax. These, as Smith very properly terms them, are fundamental characteristics. Associated with these one usually finds, also, the long, stringy neck with, as pointed out by Keith and Williams, a forward curvature of the cervical region of the spine—"ewe-neck;" there is a forward incline or drooping of the shoulders, with a chest undersized, collapsed and flattened. The upper abdomen is narrow, contracted and somewhat scaphoid with the characteristic deep epigastric angle; the lower abdomen is often prominent, pouchy and relaxed, especially in multiparous women. Within this cavity, various degrees of visceral displacement may be readily outlined, the organs most often suffering being the stomach, colon and right kidney.

In the study of these cases, one quickly comes to ask this very important question: What is the real relation between these demonstrable visceral displacements and the symptomatology presented? Truly, the thought lies near that a mechanical correction of these defects should at once relieve the situation.

Possibly so, if the relation of *cause* and *effect* could be clearly and uncompromisingly established. But is it not true that these poor sufferers often drift from one surgeon to another, each one finding some particular organ which, if, in his opinion, is "tacked up," or removed, will bring to her that much sought for something, good health—a something which, by nature, she has never enjoyed, nor ever will enjoy? No surgeon can afford to draw hasty conclusions, based on a superficial and perfunctory examination, when a patient belonging to this class presents herself. A most careful and painstaking history—both personal and family—must be had, together with an exhaustive study of her physical frame; no less important than these, also, is to obtain a correct estimate of her mental poise, whether or not she is introspective, moody, melancholic.

After this has been done, the next question to be answered is: To what extent is this retroverted uterus or this prolapsed kidney *actually* responsible for the symptomatic findings in this case? Is this woman, in truth, suffering from a sore womb or a *sore brain?* Not a few observers have tried to divide visceroptotics into two distinct classes—"congenital" and "acquired," and apportion the treatment accordingly. That is to say, for the former or congenital type the treatment should be medicinal and non-operative; while for the latter, or acquired type the treatment should be operative.

Yet, anyone who has given this subject much thought or study will soon conclude that such sharp lines of division are impractical and unsatisfactory, because of the apparent very frequent blending of both factors in the same individual.

If, after a careful weighing of all evidence which can be had, a logical conclusion can be reached that the displaced organ or organs are producing the symptoms, then the patient is entitled to and should receive such operation as will restore them to their former natural position. Quite naturally, if the case is plainly one of the acquired type—that is due to the stress and strain of a laborious life and frequent child-bearing—then the results to be had from surgical interference will be most gratifying. In any case, these patients are, as a rule, of a nervous temperament and should be safeguarded against every possible shock, both physical and mental. Of especial importance is it to see that the period of convalescence after an operation is properly conducted, and that they are not permitted to too soon take up the regular routine of their daily life.

